# Lifetime history of hypertensive disorders of pregnancy is associated with shorter sleep duration and more sleep disturbance in midlife: results from the Project Viva women’s health cohort

**DOI:** 10.1186/s13293-025-00725-4

**Published:** 2025-07-01

**Authors:** Kimia Heydari, Sheryl L. Rifas-Shiman, Suzanne M. Bertisch, Elizabeth B. Klerman, Jorge E. Chavarro, Emily Oken, Karen M. Switkowski

**Affiliations:** 1 Harvard Medical School, Boston, MA USA; 2 Department of Population Medicine, Harvard Pilgrim Health Care Institute, Harvard Medical School, 401 Park Drive Suite 401E, Boston, MA 02215 USA; 3 Division of Sleep and Circadian Disorders, Department of Medicine, Brigham and Women’s Hospital, Harvard Medical School, Boston, MA USA; 4 Department of Neurology, Massachusetts General Hospital, Boston, MA USA; 5 Division of Sleep Medicine, Harvard Medical School, Boston, MA USA; 6 Department of Nutrition, Harvard T. H. Chan School of Public Health, Boston, MA USA; 7 Department of Epidemiology, Harvard T. H. Chan School of Public Health, Boston, MA USA; 8 Channing Division of Network Medicine, Department of Medicine, Brigham and Women’s Hospital, Harvard Medical School, Boston, MA USA

**Keywords:** Hypertensive disorders of pregnancy, Midlife, Sleep duration, Sleep quality, Project Viva

## Abstract

**Background:**

Hypertensive disorders of pregnancy (HDP) are associated with worse prenatal and perinatal sleep health and higher cardiovascular disease risk beyond the peripartum period. The relationship of HDP with sleep health in midlife, when sleep problems are common, remains unclear.

**Methods:**

We studied women enrolled in Project Viva during early pregnancy (1999–2002) with sleep outcomes assessed in midlife (2017–2024). We determined lifetime HDP via medical records from the index pregnancy and self-report both at enrollment and during midlife. Outcomes were (i) self-reported sleep duration and sleep quality, using the patient-reported outcomes measurement information system sleep disturbance and sleep-related impairment instruments at mean 52.3yrs; and (ii) objectively measured 5-day sleep duration and efficiency by wrist actigraphy at mean 55.8yrs in a subset. We performed linear and logistic regression models adjusted for enrollment age, education, parity, household income, pre-pregnancy BMI, race, and ethnicity and considered modification by social determinants of health.

**Results:**

Of 767 participants, 23% had a lifetime history of HDP, 4% had ≥ 2 episodes, and 7% had HDP during their last pregnancy. Mean (SD) daily sleep duration was 7.1 (1.0) hours by self-report and 6.7 (1.0) hours by actigraphy. Any (vs. no) lifetime HDP was associated with shorter self-reported (-8 min, 95% CI: -19, 2) and actigraphy-measured (-16 min, 95% CI: -31, -1) sleep duration. Estimates were stronger but with wider CIs for those with ≥ 2 (vs. no) HDP episodes (e.g., -23 min, 95% CI: -53, 6 for actigraphy-measured sleep duration). Mean (SD) sleep disturbance T-score was 48.6 (7.4) and sleep-related impairment was 45.8 (8.5). Any lifetime HDP (vs. none) was associated with higher (worse) sleep disturbance T-score (1.85 points, 95% CI: 0.28, 3.42) with stronger associations for ≥ 2 HDP episodes (3.41 points, 95% CI: 0.17, 6.65) and for HDP in the last pregnancy (3.63 points, 95% CI: 0.70, 6.57). HDP was not associated with self-reported sleep-related impairment or sleep efficiency.

**Conclusions:**

History of HDP was associated with shorter sleep duration and higher sleep disturbance in midlife. Future work should investigate the contribution of sleep health to associations of HDP exposure with cardiovascular disease risk in later life.

**Supplementary Information:**

The online version contains supplementary material available at 10.1186/s13293-025-00725-4.

## Abstract (plain language summary)

Hypertensive disorders of pregnancy, which affect 1 in 7 pregnancies, are when a person gets high blood pressure during or soon after pregnancy. Women with high blood pressure around the time of pregnancy have more health problems, such as heart attacks, during pregnancy and into later life. Sleep problems during pregnancy may increase the chance of getting high blood pressure around pregnancy. However, it is unknown if high blood pressure during pregnancy relates to sleep in middle age. This is important because many women report sleep problems in middle age. Not getting enough or restful sleep can raise the risk of heart attacks in later life.

In this study, we tested whether having high blood pressure around pregnancy was linked to worse sleep in middle age. We used data from Project Viva, a study based in the United States that followed women for about 20 years, from early pregnancy through midlife. We found that women who had hypertensive disorders of pregnancy experienced shorter sleep duration and more sleep disturbance around ages 50–55 years. These relationships might be stronger in women who are not non-Hispanic White and who do not have a college education. We suggest clinicians ask their patients about their pregnancy history, including high blood pressure, to help inform the patient’s health risks in midlife. We also recommend future work to understand if sleep duration or sleep disturbance might explain how high blood pressure around pregnancy relates to heart disease in later life.

**Supplementary Information:**

The online version contains supplementary material available at 10.1186/s13293-025-00725-4.

## Highlights

Hypertensive disorders of pregnancy (HDP) are common and highly morbid complications that influence both short- and longer-term women’s health.

We examined associations of lifetime HDP history with midlife sleep outcomes in women enrolled in the Project Viva longitudinal cohort study. We assessed sleep outcomes by self-report in over 750 women and by actigraphy in a subset of 375 women.

We found that, compared to no lifetime history, a lifetime history of HDP was associated with a daily average of 15–20 min shorter sleep duration and greater sleep disturbance. We also found that HDP exposure during last pregnancy, and in two or more pregnancies, were more strongly associated with adverse midlife sleep health outcomes.

Social determinants of health may modify these associations, but more research is needed.

These findings support calls for assessment of lifetime history of HDP in evaluating health and disease risk in midlife and beyond.

**Supplementary Information:**

The online version contains supplementary material available at 10.1186/s13293-025-00725-4.

## Background

Hypertensive disorders of pregnancy (HDP), including preeclampsia and gestational hypertension, are common complications that affect 2–8% of pregnancies and are a leading cause of pregnancy-related morbidity and mortality worldwide [1–3]. Both preeclampsia and gestational hypertension are characterized by new-onset hypertension after the 20th week of pregnancy. Preeclampsia is additionally accompanied by additional maternal multi-system organ failure [1, 4].

The associations of HDP with longer-term health is an ongoing area of investigation, reflecting growing interest in the long-term impacts of the perinatal period in shaping women’s health [5, 6]. Most prominently, a number of studies have shown associations between HDP history and increased risks for later life cardiometabolic diseases [7]. In a US cohort study, pregnancy complications were associated with higher mortality 50 years later, with higher incidence of some complications in Black individuals, suggesting that disparities in pregnancy health have life-long implications for risk of morbidity and mortality [8, 9]. While HDPs can be managed with appropriate treatment, social determinants of health (SDoH) including financial resources, access to healthcare and social support impact access to treatment and contribute to disparities in reproductive health outcomes. Inequitable access to antihypertensive therapy as well as resources to support a healthy lifestyle during pregnancy contribute to disparities in maternal and infant health, with cascading effects on lifetime cardiovascular health [10–12].

While the mechanisms that link HDP to cardiovascular disease (CVD) risk decades later are not fully understood, sleep duration and quality may be two novel factors that mediate these associations. Evidence is robust that sleep problems, especially sleep-disordered breathing, are associated with subsequent risk for hypertension and other CVD [13, 14], likely by promoting greater inflammation, endothelial dysfunction, and increased sympathetic tone [15, 16]. Sleep-disordered breathing, including obstructive sleep apnea, is associated with the development of HDP, and the two conditions share many risk factors suggesting a common underlying mechanism [17, 18]. More limited evidence among nonpregnant adults suggests that the relationship between hypertension and sleep disturbances may be bi-directional, with hypertension disrupting circadian blood pressure rhythms and predisposing to later insomnia [19, 20]. These underlying risk factors may be further exacerbated by SDoH impacting both access to effective hypertensive treatments and lifestyle factors supporting both hypertensive control and healthy sleep. During midlife, especially in perimenopause, many women experience insomnia and disrupted sleep [21, 22]. While some predictors of sleep duration and quality among women at midlife have been thoroughly studied (e.g., obesity, sleep-disordered breathing, and vasomotor symptoms of menopause), other adverse physiologic events earlier in life, such as HDP, that may contribute to midlife sleep problems are not well-understood. Furthermore, while many studies examining longer-term health outcomes related to HDP have examined associations with complications in the first pregnancy or only one pregnancy, emerging evidence highlights the importance of considering the entire reproduction history and how multiple pregnancy complications influence associations between peripartum and midlife health. One recent study found that complications in a woman’s final pregnancy were associated with higher risk for CVD death than complications that occurred only during the first pregnancy [23].

The objective of this analysis was to investigate associations of HDP history with sleep duration and quality, including sleep disturbance, sleep-related daytime impairment, and sleep efficiency, during midlife. We studied 767 parous women participating in Project Viva, a longitudinal study of women recruited during pregnancy and subsequently followed for approximately 2 decades. We hypothesized that a history of HDP would be associated with shorter sleep duration, higher disturbance during sleep, lower sleep efficiency and higher sleep-related impairment during wakefulness at midlife. We additionally hypothesized that associations would be stronger for those with HDP complicating the last lifetime pregnancy. Given the documented higher rates of HDP among women from minority backgrounds compared with non-Hispanic White women and the proposed role of SDoH in management of HDP and mitigation of adverse outcomes, our analysis includes race and ethnicity, maternal education and annual household income at enrollment in exploring the strength of this association.

## Methods

### Study participants

**Fig. 1:**
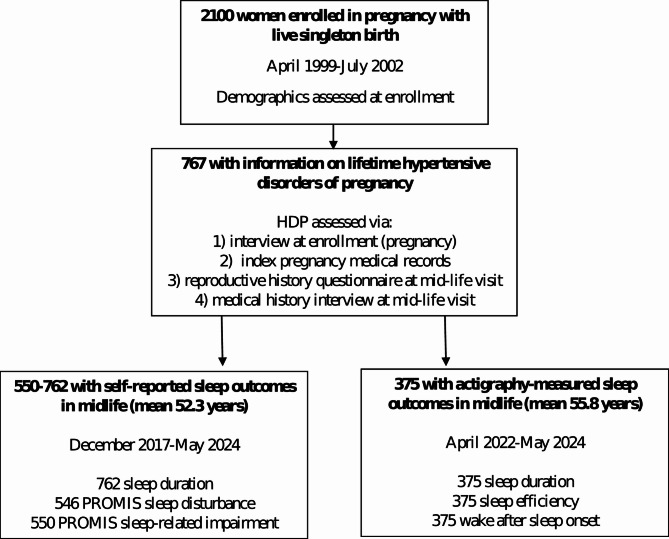
Flow diagram of participants with exposure and outcome data

Project Viva enrolled pregnant women at their initial prenatal visits at a multi-specialty group practice in eastern Massachusetts. Eligibility criteria for the cohort included enrollment prior to 22 weeks gestation, ability to communicate in English, plans to stay in the study area through delivery, and singleton gestation [24]. As described previously, we conducted in-person visits with mothers and their children in the mother’s pregnancy, at delivery, at ~ 6 months postpartum (child’s infancy), and in early and mid-childhood and early and mid-adolescence, and we administered questionnaires annually in the intervening years. The Mid Life visit was conducted concurrently with the mid-adolescence visit and mothers and their children were followed separately from that time point forward. The visits and questionnaires prior to this time point were primarily focused on collecting data relevant to the children. For this analysis, we used data collected during the enrollment pregnancy (April 1999-July 2002) and approximately 2 decades later (“midlife”, 2017–2024). Midlife data collection timepoints included the Mid-Life Visit (December 2017-August 2021), a Year 19 Questionnaire (December 2019-June 2022) and Women’s Health Visit 1 (April 2022-May 2024) [25]. Of 2100 women with live births, 767 had information on lifetime hypertensive disorders of pregnancy (Fig. 1), of whom 762 provided self-reported sleep duration or quality at the Mid-Life Visit or Year 19 questionnaire (mean age 52.3, SD 5.2 years). Additionally, 375 participated in objective monitoring of sleep with actigraphy at the Women’s Health Visit 1 (mean age 55.8 years, SD 4.8).

### Exposures

To obtain lifetime history of HDP, we collected information from four sources throughout participants’ involvement with the study. First, in the interview conducted in the first trimester of their index pregnancy at enrollment in the Project Viva cohort, research assistants asked participants whether they had been given a diagnosis of hypertension or preeclampsia in any of their past pregnancies. Second, we abstracted clinical data on blood pressure, urine protein, and diagnostic and discharge codes related to gestational hypertension or preeclampsia from both inpatient and outpatient medical records during the index pregnancy [26]. Third, during the Mid-Life Visit, participants completed a detailed reproductive history questionnaire on which they reported each of their lifetime pregnancies and any complications for each pregnancy including occurrence of “pregnancy-related high blood pressure” or “pre-eclampsia/toxemia.” Finally, we identified additional cases of HDP from a medical history interview conducted at the Mid-Life Visit which asked about any occurrence of “high blood pressure during a time when you were pregnant.”

For our analyses, we combined gestational hypertension and preeclampsia, both for reasons of power and as we were not entirely able to distinguish the two measures on our questionnaire assessments. Participants with pre-pregnancy chronic hypertension, identified based on interviews at recruitment, were classified as HDP or no HDP depending on their history of pregnancy-related hypertensive disorders identified as above. Because we defined exposure groups based on HDP status only, both the exposed and unexposed groups included participants with pre-pregnancy chronic hypertension. We examined chronic hypertension as a covariate in a sensitivity analysis.

Given evidence that CVD risk increases with more episodes of HDP as well as with HDP occurrence in the last lifetime pregnancy, we examined two categorizations of the timing of lifetime HDP in relation to later sleep outcomes [23, 27, 28]. First, we categorized lifetime HDP by the number of occurrences as 0, 1, or 2+. Second, we categorized lifetime HDP history as (i) HDP during the woman’s final pregnancy, (ii) HDP in any previous pregnancies but not the last pregnancy, or (iii) no history of HDP.

### Outcomes

#### Self-reported sleep duration and sleep quality (sleep disturbance and sleep-related impairment)

We assessed current self-reported sleep duration from the Year 19 questionnaire, or, if that was not available, from the Mid-Life Visit questionnaire, using the question “In the past month, on average, how many hours per day did you sleep in a usual 24-hour period? (Answer separately for weekdays and for weekend days).” We assessed participants’ sleep quality in the past 7 days using two of the short-form versions of the validated, publicly-available patient-reported outcomes measurement information system (PROMIS) [29] sleep bank [30, 31] on the Year 19 Questionnaire. These self-assessment instruments were developed and validated for use across the general population, those with sleep disorders, and recently, in perimenopausal women [32].

Using the PROMIS Item Bank v1.0—Sleep-Related Impairment—Short Form 4a, participants reported sleep-related impairment by answering 4 questions on a 5-point scale (ranging from not at all to very much). These questions assess whether respondents felt sleepy or experienced trouble concentrating during the daytime due to poor quality sleep. In addition, participants rated sleep disturbance in the past 7 days by answering the PROMIS Item Bank v1.0—Sleep Disturbance—Short Form 4a. This questionnaire includes 1 question about the overall quality of sleep and 3 questions about the extent to which participants found sleep to be refreshing, experienced problems with sleep, or had difficulty falling asleep on a 5-point scale with options “not at all”, “a little bit”, “somewhat”, “quite a bit” and “very much”.

#### Actigraphy-derived sleep duration and sleep efficiency

We invited participants who attended the Women’s Health Visit to wear a GENEActivVR Original actigraph (Activinsights Corporation) for 7 consecutive days and complete daily morning sleep diaries. Raw device data for participants with ≥ 5 days was uploaded and transmitted securely to the Brigham and Women’s Hospital Sleep Reading Center.

### Assessment of SDoH variables and other covariates

At the time of enrollment in Project Viva, participants reported their date of birth, education level, annual household income, prenatal smoking status, and parity. Participants self-reported race and ethnicity via a self-administered questionnaire during midlife and at enrollment; we used the midlife reports when available and if missing, reports from enrollment. We calculated pre-pregnancy BMI for the index pregnancy (kg/m^2^) from self-reported pre-pregnancy weight and height, which have been previously validated [33]. We calculated participants’ age at each visit using their date of birth and the visit date.

### Statistical analysis

The PROMIS sleep-related impairment and sleep-disturbance scales have been standardized to a population median of 50 [30]. We calculated T-scores for each measure based on this standardized scale. A higher T-score for sleep disturbance and sleep-related impairment indicates a higher symptom burden.

Upon receipt of the actigraphy data, raw plots were visually inspected for quality assurance, including verification of “off-wrist” time. The raw data were then processed using a combination of the open-source R package called GGIR [34] and self-report sleep diaries (when available as “guides”). GGIR is an open-source R package, widely used in multiple large cohorts to analyze sleep data [35], including the UK biobank and Whitehall II. GGIR uses embedded processes and algorithms to perform basic calibration, quality control, and generate epoch-level (60-second) activity data used to detect sleep and output sleep metrics. For this study, we used GGIR to implement algorithms to detect bouts of sustained inactivity [36–38], then, as available, the sleep diary was used as a “guide” to note the start and end of the sleep window. Our embedded weighted-activity algorithm applies hierarchical approach to mark sleep-wake intervals using event markers, diaries, light, and activity data and output sleep metrics, including estimated sleep duration and sleep efficiency.

We compared the distribution of participants’ characteristics and sleep data across categories of lifetime HDP status. We used percentages for categorical variables and means and standard deviations for numerical variables.

We examined associations of lifetime HDP with self-reported and actigraphy-based continuous sleep duration (minutes/night), sleep efficiency, sleep disturbance T-score, and sleep-related impairment T-score using multivariable linear regression models. We also examined associations with two dichotomous outcomes, including average nightly sleep duration (≥ 7 v. <7 h/d, selected based on American Academy of Sleep Medicine and Sleep Research Society recommendations for adults [39]) and average 7-day sleep quality (good/very good vs. fair/poor/very poor) using logistic regression models. The number of individuals with sleep duration < 6 h/d was too small for analysis (only 9% of the sample overall).

In alignment with our prior work on HDP [40], we used a parsimonious set of covariates that reflected characteristics present before the first lifetime pregnancy or prior to enrollment. We did not adjust for factors present in midlife, such as mood, menopausal status, or current BMI, as these characteristics are likely to be mediators of the relationship between HDP and sleep health and we were interested in examining the overall association. We initially ran 4 models, each building on the previous model with additional covariates to assess confounding by individual variables or sets of related variables (e.g. sociodemographic factors). The first model (Model 1) was adjusted for age at enrollment. Model 2 was further adjusted for education, parity, and annual household income at enrollment. Model 3 was adjusted for pre-pregnancy BMI at the Viva index pregnancy. Model 4 was further adjusted for race and ethnicity, which have been strongly associated with both risk for HDP and sleep health and are likely a proxy for unmeasured risk factors such as disparities in access to resources and care [41]. Because we did not observe marked differences across adjusted models, we present as primary results only the age-adjusted and fully-adjusted estimates.

To explore the impact of SDoH on the relationship between HDP and sleep outcomes, we additionally ran our models stratified by participant race and ethnicity (non-Hispanic White vs. Hispanic, Non-Hispanic Black, Non-Hispanic Asian, > 1 race or other race, as we did not have sufficient sample size to more finely characterize participants who were not non-Hispanic White), maternal education (4-year college or graduate degree vs. some college or associate’s degree, high school degree/GED, or < 12th grade completed) and annual household income at enrollment (>$70,000 vs. ≤$70,000) and calculated p-values for the interaction of HDP with each dichotomous SDoH variable. We did not include the potential effect modifier of interest as a covariate in stratified models examining effect modification by that variable.

We also conducted sensitivity analyses in which we: (1) further adjusted models for pre-pregnancy chronic hypertension, which was self-reported by participants at enrollment, and (2) excluded participants with pre-pregnancy chronic hypertension from the analysis.

We conducted all analyses using SAS Enterprise Guide, version 8.3.

## Results

Among 767 participants with information on HDP and any sleep outcome (Fig. 1) mean (SD) pre-pregnancy BMI was 24.7 (5.0) kg/m^2^ and 48% of participants were nulliparous at enrollment. Mean (SD) age at study enrollment was 32.5 (5.0) years, 52.3 (5.2) years at the time of self-reported sleep duration and PROMIS questionnaire completion, and 55.8 (4.8) years at actigraphy (Table 1). Participants predominantly identified as non-Hispanic White (69%), 14% identified as non-Hispanic Black, 6% as non-Hispanic Asian, 9% as Hispanic, and 3% as another race, including > 1 race. We identified 178 (23%) participants as having lifetime HDP; 4% of participants (32/743 with this information) had 2 or more episodes of HDP in their lifetime, and 7% (46/701) had HDP during their last pregnancy. Women with a history of HDP had higher pre-pregnancy BMI (26.6 vs. 24.1 kg/m^2^) and were more likely to identify as non-Hispanic Black (20% vs. 13%). Characteristics of the subset who participated in actigraphy were very similar to those of the overall sample (see Supplemental Table 1 provided in Additional file 1).

**Table 1:** Participant characteristics overall and by lifetime history of hypertensive disorders of pregnancy (HDP)

		Lifetime HDP		
Characteristic	Overall	No	Yes	
	*n* = 767	589 (77%)	178 (23%)	
	Mean (SD) or N (%)			*P*-Value
*HDP details*				
1	122 (16%)	--	--	
2+	32 (4%)	--	--	
Ever but not in final pregnancy	66 (9%)	--	--	
In final pregnancy	46 (7%)	--	--	
Self-reported history of high blood pressure/hypertension at enrollment	35 (5%)	14 (2%)	21 (12%)	
Age at study enrollment, years	32.5 (5.0)	32.6 (5.0)	32.3 (5.2)	0.50
Age at first lifetime pregnancy, years	28.7 (6.1)	28.8 (6.0)	28.2 (6.3)	0.24
Pre-pregnancy body mass index, kg/m^**2**^	24.7 (5.0)	24.1 (4.7)	26.6 (5.6)	< 0.0001
*Race and ethnicity*				0.06
Hispanic	67 (9%)	51 (9%)	16 (9%)	
Non-Hispanic White	525 (69%)	414 (70%)	111 (62%)	
Non-Hispanic Black	110 (14%)	74 (13%)	36 (20%)	
Non-Hispanic Asian	44 (6%)	36 (6%)	8 (4%)	
> 1 race or other	20 (3%)	13 (2%)	7 (4%)	
College graduate at study enrollment	572 (75%)	446 (76%)	126 (71%)	0.16
Nulliparous at study enrollment	371 (48%)	289 (49%)	82 (46%)	0.48
Lifetime pregnancies	3.1 (1.5)	3.2 (1.4)	2.9 (1.8)	0.11
Household income at enrollment >$70,000/year	457 (65%)	357 (66%)	100 (64%)	0.68
Self-reported history of hypertension at enrollment	35 (5%)	14 (2%)	21 (12%)	< 0.0001
*Self-reported sleep outcomes* ^**†**^				
Age at self-reported sleep duration, years	52.3 (5.2)	52.3 (5.1)	52.1 (5.4)	0.54
Average daily sleep duration, hours	7.1 (1.0)	7.2 (1.1)	6.9 (1.0)	0.02
Sleep duration < 7 h/d	259 (34%)	189 (32%)	70 (40%)	0.05
PROMIS sleep disturbance, T-score	48.6 (7.4)	48.2 (7.6)	49.9 (6.9)	0.03
PROMIS sleep-related impairment, T-score	45.8 (8.5)	45.7 (8.5)	46.3 (8.7)	0.47
Sleep quality				0.06
Very Poor	12 (2%)	9 (2%)	3 (2%)	
Poor	66 (12%)	48 (11%)	18 (15%)	
Fair	198 (35%)	145 (33%)	53 (43%)	
Good	224 (40%)	180 (41%)	44 (35%)	
Very Good	58 (10%)	52 (12%)	6 (5%)	
Sleep quality (good/very good v. very poor-fair)	282 (51%)	232 (53%)	50 (40%)	0.01
*Actigraphy-measured sleep outcomes* ^*‡*^				
Age at actigraphy, years	55.8 (4.8)	55.9 (4.6)	55.4 (5.2)	0.37
Average nightly sleep duration, hours	6.7 (1.0)	6.8 (1.0)	6.4 (1.0)	0.003
Average sleep efficiency, percent	86.9 (7.0)	87.2 (7.0)	85.9 (7.2)	0.11

Characteristics of 767 female participants in Project Viva. Data available for *n* = 743 on count of HDP pregnancies, data available for *n* = 701 on HDP in final pregnancy.^**†**^On Year 19 Questionnaire or Mid-Life Visit; ^**‡**^At Women’s Health Visit 1, *n* = 375

P-values were obtained from t-tests for continuous variables and chi-square tests for categorical/dichotomous variables

Mean (SD) self-reported midlife sleep duration among 762 women was 7.1 (1.0) h/d; it was lower among those with a history of HDP than those without (6.9 vs. 7.2 h) (Table 1). In models adjusted for age at enrollment only, any lifetime history of HDP (vs. no history) was associated with 12 fewer minutes of sleep (95% CI: -22, -1) measured by self-report (Table 2). After adjusting for additional potential confounders (maternal education, parity, annual household income at enrollment, pre-pregnancy BMI, race, and ethnicity) the estimate modestly attenuated, and the confidence interval crossed the null. Associations of HDP with self-reported sleep duration dichotomized as ≥ 7 v. <7 h/d were generally consistent with the associations of HDP with continuous sleep duration (Table 3). For example, HDP in the last pregnancy (vs. never HDP) was associated with about half the odds of sleep duration > 7 h/d in age-adjusted models (odds ratio (OR): 0.52, 95% CI: 0.29, 0.96), an association that attenuated somewhat with multivariable adjustment (OR: 0.57, 95% CI: 0.29, 1.12).

**Table 2:** Associations of lifetime hypertensive disorders of pregnancy (HDP) history with midlife sleep outcomes

HDP History	Age Adjusted	Fully Adjusted	Fully adjusted + self-reported HTN
	β (95% CI)		
*Outcome: Self-reported sleep duration*,* minutes*			
Any HDP vs. never	**-12 (-22**,** -1)**	-8 (-19, 2)	-9 (-19, 2)
1 episode of HDP vs. never	-10 (-22, 2)	-5 (-17, 7)	-6 (-18, 6)
2 + episodes of HDP vs. never	-14 (-36, 8)	-16 (-37, 6)	-17 (-39, 5)
HDP ever, not in last pregnancy vs. never	-4 (-20, 12)	1 (-14, 17)	1 (-15, 16)
HDP in last pregnancy vs. never	-13 (-32, 5)	-14 (-32, 4)	-15 (-33, 4)
*Outcome: Self-reported PROMIS sleep disturbance T score*			
Any HDP vs. never	**1.60 (0.12**,** 3.09)**	**1.85 (0.28**,** 3.42)**	**1.90 (0.30**,** 3.49)**
1 episode of HDP vs. never	1.36 (-0.41, 3.12)	1.35 (-0.51, 3.20)	1.37 (-0.51, 3.24)
2 + episodes of HDP vs. never	2.55 (-0.56, 5.67)	**3.41 (0.17**,** 6.65)**	**3.44 (0.17**,** 6.72)**
HDP ever, not in last pregnancy vs. never	0.84 (-1.42, 3.09)	0.91 (-1.50, 3.32)	0.86 (-1.56, 3.28)
HDP in last pregnancy vs. never	**2.90 (0.08**,** 5.72)**	**3.63 (0.70**,** 6.57)**	**3.55 (0.60**,** 6.51)**
*Outcome: Self-reported PROMIS sleep-related impairment T score*			
Any HDP vs. never	0.57 (-1.12, 2.26)	0.76 (-1.01, 2.52)	0.77 (-1.01, 2.56)
1 episode of HDP vs. never	0.94 (-1.05, 2.93)	0.86 (-1.21, 2.92)	0.86 (-1.23, 2.95)
2 + episodes of HDP vs. never	1.50 (-2.03, 5.03)	2.46 (-1.16, 6.08)	2.47 (-1.19, 6.13)
HDP ever, not in last pregnancy vs. never	-0.13 (-2.66, 2.40)	-0.02 (-2.70, 2.66)	0.04 (-2.65, 2.72)
HDP in last pregnancy vs. never	1.08 (-2.02, 4.18)	1.77 (-1.43, 4.97)	1.87 (-1.35, 5.09)
*Outcome: Actigraphy-measured average nightly sleep duration*,* minutes*			
Any HDP vs. never	**-22 (-36**,** -8)**	**-16 (-31**,** -1)**	**-15 (-30**,** -1)**
1 episode of HDP vs. never	-15 (-32, 2)	-9 (-26, 9)	-8 (-26, 9)
2 + episodes of HDP vs. never	**-31 (-59**,** -3)**	-23 (-53, 6)	-22 (-52, 7)
HDP ever, not in last pregnancy vs. never	-19 (-40, 2)	-16 (-38, 5)	-17 (-38, 5)
HDP in last pregnancy vs. never	-24 (-48, 1)	-13 (-39, 13)	-12 (-38, 14)
*Outcome: Actigraphy-measured average sleep efficiency*,* percent*			
Any HDP vs. never	-1.45 (-3.16, 0.26)	-1.35 (-3.18, 0.47)	-1.24 (-3.07, 0.59)
1 episode of HDP vs. never	-1.08 (-3.11, 0.96)	-0.94 (-3.11, 1.23)	-0.82 (-3.00, 1.35)
2 + episodes of HDP vs. never	-2.48 (-5.86, 0.90)	-2.60 (-6.29, 1.09)	-2.39 (-6.08, 1.31)
HDP ever, not in last pregnancy vs. never	-1.25 (-3.80, 1.31)	-1.62 (-4.35, 1.11)	-1.64 (-4.36, 1.08)
HDP in last pregnancy vs. never	-1.60 (-4.60, 1.39)	-1.20 (-4.44, 2.04)	-0.94 (-4.18, 2.30)

All models are compared with the reference category of never HDP. Fully adjusted models are adjusted for age, education, parity, household income, pre-pregnancy BMI at enrollment and race and ethnicity. Models were further adjusted for history of high blood pressure/HTN at enrollment in a sensitivity analysis. Bold font indicates results that are statistically significant (95% CI excludes the null)

**Table 3:** Associations of lifetime hypertensive disorders of pregnancy (HDP) with dichotomous midlife sleep outcomes

HDP History	Age Adjusted	Fully Adjusted*
	Odds Ratio (95% CI)	
*Outcome: Self-reported sleep duration ≥ 7 v. <7 h/d*		
Any HDP vs. never	0.71 (0.50, 1.01)	0.77 (0.52, 1.15)
1 episode of HDP vs. never	0.74 (0.50, 1.12)	0.79 (0.50, 1.24)
2 + episodes of HDP vs. never	0.55 (0.27, 1.13)	0.59 (0.26, 1.33)
HDP ever, not in last pregnancy vs. never	1.06 (0.61, 1.83)	1.12 (0.60, 2.07)
HDP in last pregnancy vs. never	**0.52 (0.29**,** 0.96)**	0.57 (0.29, 1.12)
*Outcome: Self-reported sleep quality good/very good vs. fair/poor/very poor*		
Any HDP vs. never	**0.59 (0.39**,** 0.89)**	**0.53 (0.34**,** 0.82)**
1 episode of HDP vs. never	**0.58 (0.36**,** 0.94)**	**0.56 (0.33**,** 0.95)**
2 + episodes of HDP vs. never	0.69 (0.29, 1.61)	0.52 (0.20, 1.32)
HDP ever, not in last pregnancy vs. never	0.58 (0.32, 1.08)	0.53 (0.27, 1.04)
HDP in last pregnancy vs. never	0.67 (0.32, 1.42)	0.52 (0.23, 1.19)

All models are compared with the reference category of never HDP. Fully adjusted models are adjusted for age, education, parity, household income, pre-pregnancy BMI at enrollment and race and ethnicity. Bold font indicates results that are statistically significant (95% CI excludes the null)

The overall study population (~ 550 women for PROMIS outcomes) had T-scores slightly below the normalized population T-score of 50 for both sleep disturbance (48.6 (SD 7.4)) and sleep-related impairment (45.9 (SD 8.5)), indicating less sleep disturbance and sleep-related impairment compared to the general population. Sleep disturbance and sleep-related impairment T-scores were similar among those with vs. without history of HDP (Table 1). Any experience of HDP (vs. none) was associated with higher sleep disturbance T-score (indicating more sleep disturbance) (adjusted β: 1.85 points, 95% CI: 0.28, 3.42). Associations were stronger for 2 or more episodes of HDP vs. none (adjusted β: 3.41 points, 95% CI: 0.17, 6.65) and for HDP in the last pregnancy (adjusted β: 3.63 points, 95% CI: 0.70, 6.57) (Table 2). These associations persisted or strengthened after further adjusting for chronic hypertension in a sensitivity analysis (Table 2). These results were consistent with those of analyses examining associations with the outcome of self-reported sleep quality dichotomized as good/very good vs. fair/poor/very poor. In fully adjusted models, any HDP history was associated with lower odds of reporting good/very good sleep quality (OR 0.53, 95% CI: 0.34, 0.82) (Table 3). We did not observe any associations of lifetime history of HDP (either number of episodes of HDP or HDP in last pregnancy) with self-reported sleep-related impairment (Table 2).

Among the 375 women with actigraphy-measured sleep outcomes, mean sleep duration estimated via actigraphy (averaged across all observed days) was 6.7 h/d (SD 1.0); it was lower among those with a history of HDP compared to those without HDP (6.4 vs. 6.8 h, Table 1). In models adjusted for age at enrollment only, any lifetime history of HDP was associated with 22 fewer minutes of sleep (95% CI: -36, -8) measured by actigraphy (Table 2). This estimate was attenuated but still statistically significant after adjustment for maternal education, parity, annual household income at enrollment, pre-pregnancy BMI, race, and ethnicity (Table 2), and was further attenuated after adjustment for chronic hypertension in a sensitivity analysis (Table 2). Mean actigraphy-measured sleep efficiency was 86.9 (SD 7.0)%. We did not observe any associations of lifetime history of HDP (either number of episodes of HDP or HDP in last pregnancy) with sleep efficiency measured via actigraphy (Table 2). and when excluding participants with history of chronic hypertension reported at enrollment (see Supplemental Table 2 provided in Additional file 1).

In evaluating the impact of SDoH on the association between HDP exposure and midlife sleep outcomes, we found some evidence that SDoH modified the relationship between HPD and later sleep (Table 4). In fully adjusted models, having 2 + HDP episodes (vs. none) was associated with 100 fewer minutes of actigraphy-measured average nightly sleep duration (95% CI: -171, -30) among the racially and ethnically diverse group of participants who were not non-Hispanic White, while there was no association among the non-Hispanic White participants (β: -12, 95% CI: -46, 23) (p for interaction = 0.03) (Table 4). Among racially and ethnically diverse participants who were not non-Hispanic White, having repeated HDP exposures (i.e., 2+) was associated with worse actigraphy-measured average sleep efficiency vs. never having HDP (β: -14.7, 95% CI -23.3 to -6.15), whereas no association was observed among non-Hispanic White participants (β: -0.79, 95% CI -4.81 to -3.23) (p for interaction = < 0.01) (Table 4). We also observed that among women who were not college graduates, those with 2 + episodes of HDP (vs. those with no HDP) had higher self-reported sleep-related impairment (β for PROMIS t-score: 12.95, 95% CI: 1.88 to 24.02) while no associated was observed among college-educated participants (β: 0.69, 95% CI -3.15 to 4.54) (p for interaction = 0.06) (Table 4). Finally, among participants with household income >$70,000, self-reported sleep duration was 19 min lower (95% CI: -31, -7) among those with any HDP (vs. never), but there was no association among the group with lower income (β: 6, 95% CI: -13, 26, p for interaction = 0.04) (Table 4).

**Table 4:** Associations of lifetime hypertensive disorders of pregnancy (HDP) history with midlife sleep outcomes, stratified by social determinants of health

	Non-Hispanic White		College Graduate		Annual Household Income >$70,000	
HDP History	Yes	No	Yes	No	Yes	No
**Outcome: Self-reported sleep duration, minutes**						
Any HDP vs. never	-3 (-15, 8)	-22 (-56, 12)	**-12 (-23**,** -2)**	7 (-21, 36)	**-19 (-31**,** -7)**	6 (-13, 26)
1 episode of HDP vs. never	1 (-12, 14)	-19 (-57, 18)	-10 (-22, 3)	12 (-19, 44)	**-17 (-31**,** -3)**	9 (-13, 31)
2 + episodes of HDP vs. never	-13 (-36, 10)	-1 (-69, 66)	-19 (-40, 3)	4 (-72, 80)	**-26 (-50**,** -2)**	-5 (-51, 41)
HDP ever, not in last pregnancy vs. never	5 (-12, 22)	-6 (-54, 42)	-6 (-22, 10)	26 (-19, 70)	-12 (-30, 7)	14 (-15, 43)
HDP in last pregnancy vs. never	-2 (-23, 18)	-27 (-80, 27)	-16 (-35, 3)	-1 (-51, 50)	**-23 (-43**,** -2)**	-3 (-40, 34)
***Outcome: Self-reported PROMIS sleep disturbance T score***						
Any HDP vs. never	1.44 (-0.39, 3.26)	1.91 (-3.73, 7.54)	1.40 (-0.41, 3.21)	**3.41 (0.06**,** 6.76)**	1.65 (-0.24, 3.54)	2.65 (-0.33, 5.63)
1 episode of HDP vs. never	0.59 (-1.59, 2.78)	1.61 (-4.71, 7.94)	0.70 (-1.44, 2.84)	3.47 (-0.49, 7.43)	1.00 (-1.27, 3.28)	2.40 (-0.98, 5.77)
2 + episodes of HDP vs. never	**3.85 (0.06**,** 7.64)**	0.41 (-8.94, 9.76)	2.44 (-1.15, 6.03)	**9.21 (0.62**,** 17.8)**	3.12 (-0.70, 6.94)	4.76 (-1.77, 11.3)
HDP ever, not in last pregnancy vs. never	-0.19 (-2.99, 2.62)	2.51 (-5.05, 10.1)	0.45 (-2.28, 3.17)	2.73 (-2.92, 8.38)	0.34 (-2.63, 3.32)	2.69 (-1.64, 7.03)
HDP in last pregnancy vs. never	**4.16 (0.64**,** 7.68)**	0.52 (-7.39, 8.43)	3.04 (-0.29, 6.36)	5.87 (-1.10, 12.8)	**3.66 (0.25**,** 7.07)**	3.80 (-2.46, 10.1)
***Outcome: Self-reported PROMIS sleep-related impairment T score***						
Any HDP vs. never	-0.36 (-2.31, 1.58)	6.18 (-0.26, 12.6)	0.69 (-1.25, 2.63)	0.86 (-3.55, 5.26)	0.16 (-1.87, 2.19)	1.40 (-2.10, 4.90)
1 episode of HDP vs. never	-0.36 (-2.67, 1.95)	5.71 (-1.56, 13.0)	0.76 (-1.51, 3.04)	0.62 (-4.47, 5.70)	0.82 (-1.61, 3.25)	0.54 (-3.34, 4.42)
2 + episodes of HDP vs. never	1.56 (-2.47, 5.60)	5.34 (-5.22, 15.9)	0.69 (-3.15, 4.54)	**13.0 (1.88**,** 24.0)**	-0.55 (-4.59, 3.50)	7.11 (-0.63, 14.8)
HDP ever, not in last pregnancy vs. never	-1.46 (-4.42, 1.50)	5.95 (-2.90, 14.8)	0.68 (-2.22, 3.57)	-3.52 (-10.7, 3.67)	0.94 (-2.23, 4.11)	-1.44 (-6.46, 3.59)
HDP in last pregnancy vs. never	0.82 (-2.79, 4.43)	3.58 (-5.58, 12.7)	0.36 (-3.09, 3.82)	6.71 (-2.17, 15.6)	-0.11 (-3.68, 3.47)	3.61 (-3.33, 10.5)
***Outcome: Actigraphy-measured average nightly sleep duration***,*** minutes***						
Any HDP vs. never	-15 (-31, 1)	**-46 (-90**,** -3)**	**-17 (-33**,** -1)**	-1 (-44, 42)	-18 (-37, 1)	-11 (-36, 14)
1 episode of HDP vs. never	-14 (-33, 5)	-19 (-68, 30)	-9 (-28, 9)	0 (-57, 57)	-6 (-29, 16)	-13 (-41, 14)
2 + episodes of HDP vs. never	-12 (-46, 23)	**-100 (-171**,** -30)**	-30 (-61, 2)	38 (-56, 132)	**-41 (-79**,** -2)**	11 (-40, 61)
HDP ever, not in last pregnancy vs. never	-19 (-42, 4)	-13 (-87, 60)	-18 (-41, 5)	27 (-71, 126)	-9 (-38, 20)	-31 (-66, 4)
HDP in last pregnancy vs. never	-3 (-34, 28)	-60 (-119, 0)	-15 (-45, 15)	-7 (-72, 58)	-27 (-62, 9)	12 (-28, 53)
***Outcome: Actigraphy-measured average sleep efficiency***,*** percent***						
Any HDP vs. never	-1.04 (-2.90, 0.82)	**-6.68 (-12.0**,** -1.38)**	-1.90 (-3.90, 0.09)	1.94 (-2.72, 6.60)	-0.76 (-3.12, 1.59)	-2.62 (-5.56, 0.32)
1 episode of HDP vs. never	-1.24 (-3.43, 0.95)	-4.41 (-10.4, 1.55)	-1.44 (-3.77, 0.89)	2.69 (-3.66, 9.05)	-0.05 (-2.92, 2.83)	-2.72 (-6.05, 0.60)
2 + episodes of HDP vs. never	-0.79 (-4.81, 3.23)	**-14.7 (-23.3**,** -6.15)**	-3.20 (-7.22, 0.81)	2.96 (-7.58, 13.5)	-2.33 (-7.16, 2.51)	-2.47 (-8.49, 3.55)
HDP ever, not in last pregnancy vs. never	-0.91 (-3.58, 1.76)	-7.08 (-15.9, 1.75)	-2.17 (-5.03, 0.69)	7.10 (-3.81, 18.0)	0.01 (-3.65, 3.68)	**-4.65 (-8.82**,** -0.49)**
HDP in last pregnancy vs. never	-0.16 (-3.76, 3.43)	**-7.59 (-14.8**,** -0.36)**	-1.37 (-5.10, 2.36)	0.54 (-6.64, 7.71)	-1.22 (-5.70, 3.27)	-0.62 (-5.46, 4.22)

All models are compared with the reference category of never HDP. Models stratified by participant race and ethnicity are adjusted for age, education, parity, household income and pre-pregnancy BMI at enrollment. Models stratified by education are adjusted for age, parity, household income, pre-pregnancy BMI at enrollment and race and ethnicity. Models stratified by income are adjusted for age, education, parity, pre-pregnancy BMI at enrollment and race and ethnicity. Bold font indicates results that are statistically significant (95% CI excludes the null)

## Discussion

This prospective longitudinal cohort study of relatively healthy sleepers demonstrated that a history of hypertensive disorders of pregnancy (HDP) is linked to shorter sleep duration and slightly more sleep disturbance during midlife. Poor sleep is one of the most common burdensome symptoms reported by women around the time of the menopausal transition [42], and insufficient sleep duration and poor sleep quality among midlife women are associated with poorer physical and psychological wellbeing [21]. Given the now well-established links between HDP history and cardiovascular disease (CVD) risk [43, 44], as well as between poor sleep health and CVD [45, 46], our findings suggest that sleep health should be further explored as one pathway by which HDP are associated with CVD.

We identified one prior study that assessed associations of HDP with lifestyle behaviors, including sleep duration (dichotomized as < 6 h vs. ≥6 h of sleep/night) and quality in the postpartum period. In that study, outcomes were assessed at one year postpartum and there was no difference in lifestyle behaviors between women with and without a history of HDP. As far as we are aware, our study is the first to identify a link between HDP history and midlife shorter sleep duration (measured by both self-report and actigraphy) as well as higher sleep disturbance. We did not find associations of any measures of lifetime HDP history with the other measures of sleep quality we assessed, including self-reported sleep-related impairment and actigraphy-estimated sleep efficiency. We are not aware of other studies examining these outcomes in relation to HDP.

Our finding adds to the limited literature suggesting that hypertension and sleep symptoms may have a bidirectional relationship. While there is substantial evidence supporting a higher risk of HDP among women with sleep disorders before or during pregnancy [17, 47], we are not aware of evidence from longitudinal studies that has examined HDP in relation to sleep problems later in life. There is limited evidence supporting this relationship among general adult populations. In a 2022 meta-analysis, Liu et al. [19] identified 3 studies of 13,052 participants that examined hypertension at baseline and incident insomnia [48–50]. Two studies were conducted in the USA and one in Australia, with follow-up ranging from 3 to 15 years. The pooled odds ratio for incident insomnia associated with baseline hypertension was 1.20 (95% CI: 1.08, 1.32) with low heterogeneity and no evidence for publication bias. However, two of these studies were in older adults and there was limited control for important confounders, indicating that our results are not directly comparable. As discussed below, it is likely that HDP and sleep disorders reflect common underlying pathologies and may not have a causal relationship.

The pathophysiology of HDP is characterized by an imbalance of antiangiogenic and angiogenic factors within the placenta, leading to exaggerated and persistent inflammatory vascular response and long-term endothelial dysfunction [4, 51, 52]. It remains unclear to what extent associations of HDP with later CVD are explained by shared antecedents vs. intermediate factors [53]. One analysis of a Norwegian young adult population found that much of the association between HDP and later CVD risk factors could be explained by pre-pregnancy BMI, blood pressure, and lipids [54]. While we did not have measures of preconception lipids or blood pressure in our study, adjustment for pre-pregnancy BMI made little difference. Adjustment for chronic hypertension in a sensitivity analysis strengthened associations with sleep disturbance but attenuated associations with sleep duration. Another analysis of participants in the Nurses’ Health Study found that established cardiovascular risk factors arising after pregnancy, including hypertension, hypercholesterolemia, type 2 diabetes, and overweight/obesity, explained most of the increased risk of CVD conferred by gestational hypertension (84%) and preeclampsia (57%) [55]. Of note, that analysis did not examine behavioral risk factors, including sleep, that are likely to be modifiable and may be upstream of many of these physiological risk factors.

Several plausible mechanisms could explain our findings. Lasting systemic low-grade inflammation may link HDP during pregnancy with endothelial dysfunction [56]. This vascular phenomenon has a bidirectional relationship with sleep-related problems such as obstructive sleep apnea, which in turn increases cardiovascular risk through mechanisms like oxidative stress, inflammation, and sympathetic activation [57–59]. Recent scholarship points to long-term persistence of placental dysfunction after exposure to HDP: vascular function tests have revealed the presence of vascular dysfunction months, and at times years, after the resolution of clinical symptoms of preeclampsia [60–62]. Several studies have suggested that there are sex-specific responses to poor sleep. One small study examining vascular reactivity to sleep restriction found evidence that women experienced mild inflammation in response to shortened sleep [63]. A larger, prospective study observed that poorer self-reported sleep quality at baseline was associated with 5-year increase in the inflammatory biomarker IL-6 among women after adjusting for factors including age, ethnicity, BMI and snoring [64]. Finally, a large cross-sectional study found that the inflammatory markers IL-6 and hs-CRP were associated with sleep duration among women only [65]. 

Additionally, increased production of placental antiangiogenic factors gives rise to a persistent imbalance between endothelium-derived relaxing and contracting factors, which leads to maternal endothelial dysfunction that persist years after the HDP episode [62]. As a precipitating factor for sleep-disordered breathing, the presence of endothelial dysfunction may substantiate the bidirectional relationship between sleep and cardiovascular health. In view of this evidence, exploring whether negative sleep-related outcomes at midlife in women with a history of HDP manifest earlier than poor cardiometabolic outcomes may help identify at-risk individuals earlier in their lifespan for more comprehensive preventive care. Last, sleep-disordered breathing (i.e., obstructive sleep apnea, manifested as snoring) is a risk factor for developing HDP and is bi-directionally associated with hypertension [18, 66–68]. Because repetitive sleep fragmentation due to sleep-disordered breathing impairs endothelial function, the presence of sleep-disordered breathing may underlie the association between HDP exposures and midlife sleep quality and duration. While we did not assess sleep-disordered breathing in midlife in our cohort, future research might examine this link. Other factors such as chronic conditions and mood may mediate some of the associations between HDP and sleep problems, and this presents another opportunity for future research.

Associations for HDP during the last pregnancy and HDP in 2 or more pregnancies were generally stronger than those for any HDP. These results are consistent with recent studies that have shown that complications connected to the most recent pregnancy may be a stronger predictor of CVD than complications related to the first birth, and that information on complications in all pregnancies improves risk predictions [69, 70]. For example, an analysis of Norwegian registry data found that the adjusted hazard ratio (aHR) for a combined group of pregnancy complications including preeclampsia, preterm birth, and offspring small-for-gestational age was 2.85 (95% CI, 1.93, 4.20) among women with four births and any complications only in the last pregnancy whereas if a complication occurred in the first pregnancy only, the aHR was 1.74 (95% CI: 1.24, 2.45) [23]. Our results should be considered preliminary yet add to this recent literature suggesting that complications during later pregnancies may merit additional consideration when evaluating overall CVD risk.

We observed some evidence that the relationship between HDP and later sleep outcomes might depend on SDoH variables (race and ethnicity, education level, household income). As expected, the associations were stronger among participants who were not non-Hispanic White and those without a college education. Women with access to more resources and who experience less discrimination and stigma within the healthcare system may be better equipped to manage HDP, mitigating the detrimental effects on their later health [71–73]. However, these results should be interpreted with caution given the small sample sizes within many of these strata.

Limitations of this study merit consideration. Since the sample size of participants was relatively small, we were limited in our ability to assess interactions of HDP with race and ethnicity on sleep-related outcomes. There was some evidence that associations were stronger among women who were not non-Hispanic White, yet this should be interpreted with caution given the small samples sizes and our inability to parse out individual racial and ethnic groups within this diverse subset. Since both the occurrence of HDP and worse sleep outcomes are more prevalent among Black individuals than other racial and ethnic groups, likely due to socioeconomic disparities and persisting structural discrimination limiting access to early interventions for pregnancy complications, exploring the existence of this association among women different races and ethnicities merits further research [41, 74, 75]. Further, given our overall modest sample size, only 25 participants had 2 or more episodes of HDP. Given that we observed evidence of poorer sleep quality among this group, additional prospective studies with larger sample sizes of women with a history of HDP are needed to clarify relationships of this pregnancy complication with sleep outcomes. There may be other potential confounders of these relationships that we were not able to adjust for, such as sleep-disordered breathing. We also did not have information on sleep disorders before, during or shortly after pregnancy and were not able to account for long-term sleep problems in our analysis. Since the Project Viva participants are generally well educated, and all had health insurance and healthcare at enrollment, generalizability of our results may be limited. However, the cohort’s demographic characteristics were similar to that of the source population in the greater Boston area [24], and prevalence of HDP was similar to expectations for general populations.

Given our cohort participants were on average in their fifties and few reported CVD at the time of outcome assessment, we are not able to currently study associations of HDP with CVD, or the extent to which sleep health mediates these relationships. However, sleep health is an inherently important outcome to study, given the high prevalence of sleep problems among midlife women, and the importance of adequate sleep duration and quality for cognitive function, mental health, and wellbeing.

Strengths of the study included its well characterized study population. Given our comprehensive, detailed assessment of pregnancy history, we were able to examine not only the index pregnancy, but also lifetime count of HDP as well as HDP complicating the woman’s final pregnancy. Thus, we support the recent calls for studies including a woman’s complete reproductive history in understanding chronic disease risks related to HDP and other pregnancy complications [53, 70]. We also assessed history of chronic hypertension at enrollment, and after excluding participants who reported pre-pregnancy chronic hypertension in a sensitivity analysis, we observed similar associations of HDP history with midlife sleep outcomes. Though the 95% CIs for the effect estimates were wider due to the smaller sample size, these results suggest that the observed associations of HDP with midlife sleep outcomes are not explained by chronic hypertension. In addition, by relying on both self-reported as well as actigraphy-estimated data regarding sleep duration and quality, we more comprehensively assessed sleep health outcomes compared with a similar prior study, which used only self-report. We were also able to adjust for several potential confounders of the association between HDP and later sleep health. Finally, we observed less sleep disturbance and sleep-related impairment among our sample as compared to the general population, suggesting overall healthy sleep patterns. This may indicate that other risk factors contributing to poor sleep, including potential confounders of the association between HPD and sleep, were not prevalent in our population. This highlights the need for additional research on this topic, particularly in study populations with more limited resources and less access to healthcare. However, our observation that HDP may be associated with later sleep problems even in an overall healthy population suggests that reproductive histories are important to consider in evaluating health risks among all women, particularly those disadvantaged by SDoH.

### Perspectives and significance

Identification of history of HDP presents an opportunity to use women’s full reproductive history to identify women at risk for cardiovascular disease before the manifestation of disease sequelae. Given that sleep is an important marker for overall health and may mediate associations between HDP exposure and increased midlife cardiovascular disease risk, further inquiry into mechanisms underlying associations between HDP exposure and midlife sleep is necessary. Practical implications of this study include support for the recommendation that clinicians obtain comprehensive pregnancy histories from female patients to better assess risk for developing cardiometabolic disease later in life [76].

## Conclusion

In this study, lifetime history of hypertensive disorders of pregnancy was associated with higher self-reported sleep disturbance and lower sleep duration in midlife. Associations were strongest for HDP complicating the last lifetime pregnancy. While there exists a well-documented association between HDP and CVD morbidity, few studies examine associations between lifetime history of HDP with sleep, despite emerging evidence that there is a bidirectional relationship between hypertension and sleep disorders. Further research is needed to better understand potential mechanisms that link HDP with midlife sleep duration and quality and to understand sleep health as a potential risk marker for cardiovascular health. In addition, long-term studies are needed to assess to what extent prevention techniques will prove effective for reducing risk of impaired sleep and later CVD in women with a history of HDP.

## Electronic supplementary material

Below is the link to the electronic supplementary material.

Supplementary Material 1: Tables providing supplementary data: Description of data: Supplemental tables presenting participant characteristics overall and among the actigraphy subgroup (Supplemental Table 1) and associations of lifetime hypertensive disorders of pregnancy (HDP) history with midlife sleep outcomes after excluding participants with a self-reported history of chronic hypertension at enrollment (Supplemental Table 2)

## Data Availability

Data included in this manuscript are not publicly available because Project Viva’s historic consents did not allow for public data sharing. In accordance with Project Viva policies, datasets are available upon reasonable request via the Project Viva ROADMaP portal at: https://vivaroadmap.net/users/sign_up? invitation_code=welcome-to-viva-roadmap. All data collection instruments are also available via the ROADMaP.
