# Supplementary material for: Lifetime history of hypertensive disorders of pregnancy is associated with shorter sleep duration and more sleep disturbance in midlife: results from the Project Viva women’s health cohort
Source: Biol Sex Differ. 2025 Jul 1;16:46. doi: 10.1186/s13293-025-00725-4 (PMC12219990; doi:10.1186/s13293-025-00725-4)
Supplement: Supplementary file 1 — Supplementary Material 1: Tables providing supplementary data: Description of data: Supplemental tables presenting participant characteristics overall and among the actigraphy subgroup (Supplemental Table 1) and associations of lifetime hypertensive disorders of pregnancy (HDP) history with midlife sleep outcomes after excluding participants with a self-reported history of chronic hypertension at enrollment (Supplemental Table 2) [file 13293_2025_725_MOESM1_ESM.docx]

| Characteristic | **Overall** | **Actigraphy subgroup** |
| --- | --- | --- |
|  | n=767 | n=375 |
|  | **Mean (SD) or N (%)** | |
| Lifetime HDP = Yes | 178 (23%) | 84 (22%) |
| *HDP details* |  |  |
| 1 | 122 (16%) | 56 (15%) |
| 2+ | 32 (4%) | 18 (5%) |
| Ever but not in final pregnancy | 66 (9%) | 34 (10%) |
| In final pregnancy | 46 (7%) | 24 (7%) |
| Age at study enrollment, years | 32.5 (5.0) | 33.2 (4.7) |
| Age at first lifetime pregnancy, years | 28.7 (6.1) | 29.6 (5.8) |
| Pre-pregnancy BMI, kg/m^2^ | 24.7 (5.0) | 24.3 (5.0) |
| *Race and ethnicity* |  |  |
| Hispanic | 67 (9%) | 30 (8%) |
| NH White | 525 (69%) | 267 (71%) |
| NH Black | 110 (14%) | 41 (11%) |
| NH Asian | 44 (6%) | 28 (7%) |
| > 1 race or other | 20 (3%) | 9 (2%) |
| College graduate | 572 (75%) | 318 (85%) |
| Nulliparous at study enrollment | 371 (48%) | 194 (52%) |
| Lifetime pregnancies | 3.1 (1.5) | 3.1 (1.5) |
| Lifetime live births | 2.3 (1.0) | 2.3 (1.0) |
| Household income >$70,000/year | 457 (65%) | 248 (69%) |
|  |  |  |
| Self-reported history of high blood pressure enrollment | 35 (5%) | 18 (5%) |
| *Self-reported sleep outcomes^†^* |  |  |
| Age at Year 19 Questionnaire, years | 52.9 (5.0) | 53.3 (4.8) |
| Age at self-reported sleep duration, years | 52.3 (5.2) | 53.1 (4.8) |
| Sleep duration, h/d | 7.1 (1.0) | 7.2 (0.9) |
| PROMIS sleep disturbance, T-score | 48.6 (7.4) | 48.0 (7.5) |
| PROMIS sleep-related impairment, T-score | 45.8 (8.5) | 45.3 (8.3) |
| Sleep quality |  |  |
| Very Poor | 12 (2%) | 3 (1%) |
| Poor | 66 (12%) | 42 (13%) |
| Fair | 198 (35%) | 112 (34%) |
| Good | 224 (40%) | 136 (41%) |
| Very Good | 58 (10%) | 39 (12%) |

**Supplemental Table 1:** Participant characteristics overall and among the actigraphy subgroup

**Supplemental Table 2**: Associations of lifetime hypertensive disorders of pregnancy (HDP) history with midlife sleep outcomes excluding participants with self-reported history of high blood pressure at enrollment

| **HDP History** | Age Adjusted | Fully Adjusted |
| --- | --- | --- |
|  | β (95% CI) | |
| *Outcome: Self-reported sleep duration, minutes* |  |  |
| Any HDP vs. never | -8 (-19, 3) | -6 (-16, 5) |
| 1 episode of HDP vs. never | -8 (-20, 5) | -4 (-16, 8) |
| 2+ episodes of HDP vs. never | -6 (-31, 18) | -9 (-33, 15) |
| HDP ever, not in last pregnancy vs. never | -3 (-19, 14) | 1 (-15, 17) |
| HDP in last pregnancy vs. never | -7 (-28, 13) | -8 (-27, 12) |
| *Outcome: Self-reported PROMIS sleep disturbance T score* |  |  |
| Any HDP vs. never | 1.56 (-0.02, 3.14) | **1.96 (0.30, 3.62)** |
| 1 episode of HDP vs. never | 1.32 (-0.56, 3.20) | 1.39 (-0.58, 3.36) |
| 2+ episodes of HDP vs. never | 2.17 (-1.27, 5.61) | 3.52 (-0.07, 7.11) |
| HDP ever, not in last pregnancy vs. never | 0.60 (-1.76, 2.95) | 0.68 (-1.85, 3.20) |
| HDP in last pregnancy vs. never | 2.67 (-0.36, 5.70) | **3.68 (0.52, 6.84)** |
| *Outcome: Self-reported PROMIS sleep-related impairment T score* |  |  |
| Any HDP vs. never | 0.00 (-1.77, 1.78) | 0.44 (-1.41, 2.28) |
| 1 episode of HDP vs. never | 0.43 (-1.66, 2.53) | 0.52 (-1.66, 2.69) |
| 2+ episodes of HDP vs. never | 0.37 (-3.48, 4.22) | 1.97 (-2.01, 5.96) |
| HDP ever, not in last pregnancy vs. never | -0.17 (-2.80, 2.45) | 0.10 (-2.70, 2.90) |
| HDP in last pregnancy vs. never | 0.16 (-3.16, 3.47) | 1.18 (-2.24, 4.61) |
| *Outcome: Actigraphy-measured average nightly sleep duration, minutes* |  |  |
| Any HDP vs. never | -14 (-29, 1) | -13 (-28, 2) |
| 1 episode of HDP vs. never | -12 (-29, 5) | -10 (-27, 8) |
| 2+ episodes of HDP vs. never | -8 (-37, 22) | -7 (-38, 24) |
| HDP ever, not in last pregnancy vs. never | -16 (-37, 4) | -16 (-37, 6) |
| HDP in last pregnancy vs. never | -4 (-31, 22) | 1 (-27, 28) |
| *Outcome: Actigraphy-measured average sleep efficiency, percent* |  |  |
| Any HDP vs. never | -0.83 (-2.57, 0.92) | -1.07 (-2.92, 0.78) |
| 1 episode of HDP vs. never | -0.67 (-2.73, 1.38) | -0.91 (-3.12, 1.30) |
| 2+ episodes of HDP vs. never | -0.88 (-4.47, 2.70) | -1.07 (-4.92, 2.79) |
| HDP ever, not in last pregnancy vs. never | -0.54 (-3.04, 1.97) | -1.09 (-3.80, 1.63) |
| HDP in last pregnancy vs. never | -0.40 (-3.55, 2.75) | -0.31 (-3.70, 3.07) |

All models are compared with the reference category of never HDP. Fully adjusted models are adjusted for age, education, parity, household income, pre-pregnancy BMI at enrollment and race and ethnicity. Bold font indicates results that are statistically significant (95% CI excludes the null).
